# Resilient structure of nature‐based extension programs facilitates transition to online delivery and maintains participant satisfaction

**DOI:** 10.1002/ece3.6860

**Published:** 2020-10-27

**Authors:** Martin B. Main, Holly K. Ober, Shelly A. Johnson

**Affiliations:** ^1^ Department of Wildlife Ecology and Conservation University of Florida Gainesville FL USA; ^2^ UF/IFAS Extension University of Florida Gainesville FL USA

**Keywords:** blended programming, COVID‐19, curriculum, evaluation, natural resources, outreach

## Abstract

Restrictions on public gatherings in early 2020 due to the COVID‐19 pandemic resulted in cancelation of in‐person outreach programs offered by the Florida Master Naturalist Program and Natural Areas Training Academy, two successful University of Florida extension programs that provide natural history and resource management training to lay and professional audiences. In response, both programs rapidly transitioned to blended or 100% online educational methods to continue offering courses and maintain program operations. To assess participant responses to these changes, we used surveys and course registry data to evaluate and compare course enrollment, satisfaction, and outcomes among courses with new online formats to courses offered prior to the COVID‐19 pandemic. We also examined logistical challenges and key programmatic elements that facilitated the transition of both programs to increased reliance on online education. Course participants responded favorably to classes offered online. Our results revealed an audience exists for online programming, that satisfaction with online courses was high and comparable to that measured for in‐person courses, and that online approaches effectively transferred knowledge and promoted behavior change in participants. The transition to online programming required investments of time, energy, and in some cases, direct costs. However, this transition was greatly facilitated by the existence of well‐defined program protocols, educational curricula, strong partnerships, and feedback mechanisms for both programs. Long‐term investments in program structure, partnerships, and support systems enabled both programs to be resilient and adaptable and successfully implement online programming in response to the COVID‐19 pandemic.

## Graphical abstract

In response to the COVID‐19 pandemic, the Florida Master Naturalist Program and Natural Areas Training Academy transformed traditional in‐person field courses into blended and 100% online formats. We compared course enrollment, satisfaction, and outcomes between in‐person and online course formats and discuss logistical challenges and programmatic elements that enabled both programs to be resilient and successful in adapting courses to online delivery.

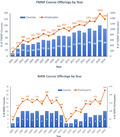

## 1 INTRODUCTION

“It is not the strongest of species that survives, nor the most intelligent that survives. It is the one that is the most adaptable to change.” Charles Darwin

The rapid spread of the COVID‐19 virus in the United States (Roser et al., 2020) necessitated changes in nearly every aspect of public life, including educational programming and public gatherings (Boeren et al., 2020; Fernandez & Shaw, 2020). University and government mandates for social distancing forced rapid revisions to traditional methods of delivering educational training to incorporate online educational methods (O'Leary, 2020). The University of Florida (UF) has a long history of providing natural history and resource management training as outreach extension programs through the Florida Master Naturalist Program (FMNP; www.MasterNaturalist.org) and the Natural Areas Training Academy (NATA; https://programs.ifas.ufl.edu/nata/). Both programs provide curricula‐based training, certificates of achievement valuable as evidence for skill development, and originally were offered only as multiday in‐person workshops that included combinations of classroom and outdoor educational experiences.

Each of these nature‐based outreach training programs adapted quickly during spring of 2020 to accommodate the new challenges brought about by the COVID‐19 pandemic. The FMNP transitioned from the use of a 100% in‐person format to a continuum of formats blending online and in‐person delivery and NATA transitioned from a blended online/in‐person format to 100% online delivery. After experimenting with these new approaches during what we refer to as the “early COVID period” (March 16‐June 30, 2020), we examined whether differences existed between this period versus our prior year programs in terms of course participants, satisfaction, and outcomes. We also discuss our experiences, challenges, and successes and conclude with recommendations for others who are considering transitioning their outreach extension programs to online formats.

## 2 PROGRAM OVERVIEWS

### 2.1 Florida Master Naturalist Program

The need to effectively engage broad audiences in conservation education has been articulated as critical to creating an environmentally literate public (Brewer, 2001) and generating support for conservation policies (Johns, 2003; Redford & Sanjayan, 2003). With these goals in mind, the Florida Master Naturalist Program (FMNP) was developed to enhance understanding of Florida's natural history and conservation issues and develop interpretive skills to effectively share that information with others (Main, 2004). Since the first courses in 2001, the FMNP has expanded from three core courses that discuss Florida's coastal, freshwater, and upland environments to include courses that teach technical skills such as wildlife monitoring and habitat evaluation and delve deeper into topics such as conservation and restoration science (Appendix S1).

Participants in the FMNP include both laypersons and professionals interested in learning more about Florida's natural resources and seeking to develop skills to become more effective as volunteers, educators, ecotourism guides, consultants, natural resource managers, and proponents for conservation. FMNP courses are taught by a statewide network of certified instructors who apply, are vetted, and go through instructor orientation prior to teaching courses. Although the FMNP is a UF extension program, courses are taught by teams of instructors that include and may be led by individuals from other agencies and organizations. These external partnerships have expanded FMNP instructor expertise and increased course quality.

The FMNP educational model uses multiple learning strategies that include classroom, field, and experiential learning opportunities such as speaking requirements and group final projects (Knowles, 1980). Interest and participation in FMNP training has increased over time (Appendix S2). During 2018 and 2019, an average of 95 FMNP courses was taught and 1,465 FMNP graduate certificates were issued annually ($\overset{-}{x}$ = 50,068 contact hours of instruction/year). Fluctuations in numbers of annual FMNP courses and graduates occur for various reasons, including hurricanes and more recently, the COVID‐19 pandemic.

### 2.2 Natural Areas Training Academy

This training program provides instruction that prepares professional land managers to sustainably manage natural areas. The impetus for Natural Areas Training Academy (NATA) was a series of state land acquisition programs spanning the past 40 years that established ~2.6 million acres in conservation lands (FDEP, 2020). NATA was initiated by The Nature Conservancy (TNC) in 2000 to train new hires in agencies and organizations tasked with the responsibility of managing these lands (Colverson & Demetropoulos, 2010; Appendix S1). Ownership of the program was transferred from TNC to UF Extension in 2008.

Participants in NATA are primarily professional land managers from public and private organizations, but also include consultants, fire practitioners, environmental educators, interns, and university students. Instructors are professional land management experts from a variety of agencies and organizations, including UF. The majority of instructors have served for many years.

The format of NATA courses during 2000–2011 was 100% in‐person for multiple days. In response to declining enrollment 2009–2011, a new partially online and partially in‐person blended learning format was initiated in 2012. This allowed for a reduction in registration fees and surveys of participants indicated they appreciated the flexibility provided by the blended approach, especially the reduction in time they needed to spend away from their job and home life (Friedl et al., 2015). Both traditional NATA courses (2000–2011) and blended courses (2012–2019) employed 3 types of learning strategies: lectures from experts, data collection during field trips, and group exercises to gain experience using new tools and practice new skills. In addition, the online component of blended course offerings was comprised of prerecorded lecture videos, quizzes, and assignments.

Natural Areas Training Academy has consistently trained an average of 130 land managers per year for the past 20 years (Appendix S2). Over 1,600 people have attended a NATA course during this time period, and 166 have earned a Certificate in Natural Areas Management (conferred when a series of 4 courses has been completed). During 2018 and 2019, 14 NATA courses were delivered by 40 instructors to 375 participants ($\overset{-}{x}$ = 4,840 contact hours of instruction/year).

## 3 PROGRAM TRANSITION TO ONLINE FORMATS

The implementation of COVID‐19 precautions required cancelation of FMNP courses (*n* = 17) scheduled during March‐June 2020 and NATA courses (*n* = 2) scheduled during April 2020. All course registrants were offered a full refund or credit for a future course. In response to course cancelations and newly instated COVID‐19 safeguards, both programs immediately initiated efforts to transition courses into new online, distance‐learning formats that included synchronous, asynchronous, and self‐directed learning activities. This included two FMNP courses that were ≥50% completed by March 16, 2020. Both courses transitioned to online formats and were completed and, although course participants were offered the option to withdraw with credit for a future course, 78% (*n* = 27) chose to complete the courses. There were no NATA courses in progress during March, but one course had been open for registration and was restructured from a hybrid to a fully online format. Registrants were offered a refund but 75% (*n* = 12) chose to take the course in the online format.

In response to COVID‐19 guidelines issued during March, the FMNP adopted a mixed model for course offerings that included a blended online and in‐person approach that required instructors schedule experiential learning activities at future dates, and a 100% online approach that required participants complete self‐directed field activities for discussion during online group meetings. FMNP instructors, therefore, provided classroom presentations, discussions and participant reporting sessions as synchronous online learning activities, assignments such as recorded webinars and videos as asynchronous learning activities, and a spectrum of options that ranged from scheduling future in‐person meetings to assigning self‐directed activities to ensure participants satisfied experiential learning requirements. The FMNP registration page posted course expectations and complete agendas for all FMNP courses.

Natural Areas Training Academy was already implementing online delivery of course materials when COVID‐19 precautions were implemented, having shifted from fully in‐person courses to a blended online and in‐person course format in 2012. Consequently, NATA was well‐positioned to transition directly from the blended format to a 100% online format. The NATA director and workshop coordinator met with instructors of each course multiple times to come to agreement on what material should be prioritized during live sessions versus what could be covered through assignments. To compensate for experience that would have been gained during field trips, a slide show was created showing photographs of the locations visited during each trip and narrated live by the same experts that would normally have led the field trip in person. NATA courses offered during COVID restrictions consisted of asynchronous online learning (prerecorded lectures, quizzes, and assignments), synchronous online learning (live lectures and group exercises) as well as postclass assignments that could be completed either alone or in pairs. Agendas for each live session were carefully planned to encourage participant involvement and avoid boredom.

## 4 EVALUATION OF ONLINE PROGRAMMING

We used program surveys and course registry data to evaluate and compare course enrollment, satisfaction, and outcomes among FMNP and NATA course participants for courses offered prior to the COVID‐19 pandemic (pre‐COVID period, 2019 and earlier) and during the early COVID period (March 16 through June 30, 2020) when in‐person events were prohibited (IRB protocols #202001365 and #201800987). Our questions of interest were whether there were differences between the pre‐COVID and early COVID periods in (a) participants who enrolled in courses, (b) participant satisfaction with courses, and (c) outcomes of courses. We also evaluated our experiences related to logistical challenges and identified key programmatic elements we found helpful for adapting FMNP and NATA training courses to include increased levels of online delivery.

### 4.1 Enrollment

We observed a strong positive response to the new online formats for courses offered by FMNP and NATA, with higher enrollment rates documented for courses offered during the early COVID period than during the pre‐COVID period for both programs (Table 1). Registrants for early COVID period courses included persons who had taken courses in pre‐COVID formats and roughly 75% of total registrants indicated COVID‐19 safety concerns and social distancing requirements did not influence their decision to enroll in the new online formats for both programs (Table 1). These decisions did not appear to be strongly influenced by occupation, because occupations of FMNP and NATA participants were similar for pre‐COVID and early COVID courses, with the exception of an influx of interns for early COVID NATA courses (Table 1). Collectively, therefore, these data reveal an audience exists that has both interest and willingness to enroll in courses with online formats offered by the FMNP and NATA regardless of the availability of courses taught as traditional in‐person courses.

**TABLE 1:** Participant enrollment, satisfaction, and outcome measures for pre‐ and early COVID courses completed by the Florida Master Naturalist Program (FMNP) and Natural Areas Training Academy (NATA)

Participant metric	Florida Master Naturalist Program		Natural Areas Training Academy	
	Pre‐COVID period Dates: 2019^a^ Courses: 89 Participants: 1,368^b^	Early COVID period Dates: Mar 16‐Jun 30, 2020 Courses: 8 Participants: 125	Pre‐COVID period Dates: 2015–2019 Courses: 10 Participants: 192	Early COVID period Dates: Mar 16‐Jun 30, 2020 Courses: 2 Participants: 70
Enrollment rate^c^	$\overset{-}{x}=76\%\left(SD=27\right)$	$\overset{-}{x}=84\%\left(SD=24\right)$	$\overset{-}{x}=62\%\left(SD=24\right)$	$\overset{-}{x}=100\%\left(SD=0\right)$
Return enrollment^d^	62%	63%	40%	23%
Influence of COVID−19 on motivation to take online courses^e^	NA	73% no influence 14% motivated by COVID−19 13% unsure	NA	76% no influence 15% motivated by COVID−19 9% unsure
Occupation	8% natural resource management or research 8% environmental educator 6% ecotourism guide 4% teacher (K‐12) 39% retired 34% other	5% natural resource management or research 9% environmental educator 7% ecotourism guide 10% teacher (K‐12) 35% retired 34% other	83% natural resource management or research 3% environmental educator 6% student/intern 2% fire fighter 5% other	54% natural resource management or research 9% environmental educator 27% student/intern 4% fire fighter 7% other
Satisfaction^f^	80% excellent 17% very good 3% good 1% fair	72% excellent 23% very good 4% good 1% fair	50% exceeded 49% fulfilled 1% not met	49% exceeded 51% fulfilled
Course format preferences^g^	NA	88% likely to enroll in 100% online courses 75% likely to enroll in blended online/in‐person courses 67% likely to enroll in 100% in‐person courses	NA	27% prefer 100% online 42% prefer blended 15% prefer 100% in‐person 15% no preference
Likelihood of recommending course to others^h^	95% yes 4% undecided 1% no	96% yes 3% undecided 1% no	98% yes 2% no	97% yes 3% no
Knowledge gain^i^	$\overset{-}{x}=44\%\left(SD=25\%\right)$	$\overset{-}{x}=51\%\left(SD=17\%\right)$	99% of participants	100% of participants
Behavior change^j^	63% reported using FMNP information for work as employee or volunteer 68% adopted personal stewardship behaviors	NA	85% plan to change the way they manage natural resources 81% expect to use agency resources more efficiently	96% plan to change the way they manage natural resources 92% expect to use agency resources more efficiently

Note

Pre‐COVID comparison dates include different years and sample sizes for FMNP and NATA. Survey dates and response rates deviate from pre‐ and early COVID headings for some metrics as noted in table.

^a^ FMNP pre‐COVID survey data from other years (2018) are noted in table with respondent sample size.

^b^ Participants represent total numbers of persons enrolled in FMNP and NATA courses; survey response rates for pre‐ and early COVID period were 79% (*n* = 1,084) and 77% (*n* = 96) for FMNP and 66% (*n* = 127) and 47% (*n* = 33) for NATA, respectively.

^c^ Enrollment rate represents percent capacity defined as the number of individuals registered divided by maximum capacity for a course * 100.

^d^ Return enrollment defined as percentage of individuals who completed a previous course (FMNP 2019 data: 29 courses, 443 respondents).

^e^ Participants (FMNP and NATA) asked, “Would you have taken this course in the online format if we were not currently in the midst of the COVID‐19 health crisis requiring social distancing?”.

^f^ Course satisfaction metrics for FMNP early COVID‐19 courses refer to the online portion of the course.

^g^ FMNP asked participants how likely they would be to enroll in future courses with 100% online, blended, or 100’% in‐person formats using a Likert scale; results represent combined “extremely likely” and “likely” responses for each category (multiple questions; results do not total 100%); NATA asked participants to identify their most preferred format for future courses (one question; results total 100%).

^h^ FMNP data from 2018 survey (*n* = 1,311 responses); results represent combined Likert scale responses (extremely likely/likely = “yes” and unlikely/extremely unlikely = “no”) regarding likelihood of recommending FMNP courses to others.

^i^ FMNP pre‐ and post‐test averages (early COVID: *n* = 3 courses); NATA participants' self‐reported gain in knowledge.

^j^ FMNP data obtained from 2018 survey (*n* = 1,305 responses).

### 4.2 Satisfaction

Participants indicated a high degree of satisfaction from courses offered by the FMNP and NATA both before and during the COVID‐19 pandemic (Table 1). The response to online courses was highly positive and similar to courses offered during the pre‐COVID period for both programs, with >95% of participants rating their experience as excellent or very good (FMNP) or as having exceeded or met expectations (NATA). The one person who indicated they would not recommend an online FMNP course to others commented “Only because I do not think the spirit of the course is the same with the online version.” The likelihood that early COVID period course participants would recommend taking an online course to others was also highly positive and similar to pre‐COVID periods with >95% of course participants responding in the affirmative for both programs (Table 1).

### 4.3 Outcomes

Knowledge gain was measured differently by the FMNP and NATA programs, but both programs reported knowledge gain among participants for both pre‐ and early COVID periods (Table 1). Although knowledge gain measured from pre‐ and post‐tests was higher for FMNP courses taught online, meaningful comparisons between in‐person and online formats may be premature because of the smaller participant sample size for online courses. What is evident is that both FMNP and NATA online formats were effective in transferring knowledge to participants.

Behavior change was also measured differently between programs. Data from postcourse surveys of FMNP graduates used to evaluate changes in behaviors were available for the pre‐COVID period but not for the early COVID period due to the short timeframe. Participants in NATA were asked about their intent to change behaviors and data from both pre‐COVID and early COVID periods indicate strong intent to change the way they manage natural resources and utilize agency resources more efficiently to do so (Table 1).

### 4.4 Logistical challenges and programmatic elements contributing to program transition

Restrictions on public gatherings in response to the COVID‐19 pandemic resulted in multiple course cancelations during March–June 2020 for both programs and substantial lost revenues and expenditures associated with processing refunds. These cancelations necessitated a rapid transition to online formats that presented both logistical challenges and costs. Although it is beyond the scope of this paper to provide a detailed cost analysis, information we provide based on our experiences may help other programs plan for similar logistical challenges and expenses when adapting existing programs to online formats.

In terms of costs, both programs made substantial investments of time and energy discussing and developing instructional policies and course guidelines that would be acceptable to instructors, appealing to participants, and would continue to meet established program requirements. There was also time and direct expense associated with modifying program policies and support mechanisms associated with registration, mailing, websites, and marketing. In some cases, policy changes resulted in increased direct expenses, such as the need to mail course materials directly to participants rather than mailing in bulk to instructors to distribute to participants during the first class.

Elements that positively contributed to the transition to online programming included having established curricula, educational materials, and defined course expectations for both programs. Although detailed curricula were originally developed to ensure consistency in course content among dates, locations, and instructors, these standardized course materials also proved to be beneficial when converting courses to online delivery and made presenting educational components online relatively straightforward. Another important factor was the existence of long‐term relationships with program instructors and partners who provided advice and logistical support. The strong partnerships with program instructors was especially important because instructors assisted in the transition to online programming by providing suggestions and contributing time and effort to make online programming a positive experience for participants. The third major element that contributed to a successful transition and high levels of enrollment for online courses was the reputations of both the FMNP and NATA as excellent educational programs, as evidenced by survey results indicating high levels of course satisfaction and the likelihood that participants would recommend courses to others (Table 1).

## 5 RECOMMENDATIONS FOR PROGRAM TRANSITION TO ONLINE TRAINING

Our experiences implementing a rapid transition to new online formats in response to the COVID‐19 pandemic provide several key insights that may be of value to programs considering similar changes. Although we recognize there are many factors that could be important, we identified the following as key elements to our success:

- Program structure—well‐defined program protocols for FMNP and NATA, including participant and instructor course requirements and expectations ensured program structure remained consistent when transitioning to online programming.
- Educational curricula and materials—the existence of educational curricula and supporting materials (e.g., presentations and manuals) assisted instructors with transitioning to online delivery of classroom components and maintaining course consistency goals; the freedom for instructors to supplement existing materials with online learning opportunities added new and creative elements to courses.
- Logistics—strong partnerships with instructors, participants, and support organizations and dedicated program personnel contributed to successful transitions and high degrees of satisfaction among participants in response to new online formats.
- Feedback mechanisms—strong lines of communication with instructors and feedback from students provided information used to fine tune program elements.

We have few negatives to report regarding our transitions to blended and 100% online programming. The time, effort, and expense needed to think through and implement online course offerings were unavoidable consequences of the COVID‐19 pandemic and represented short‐term investments necessary to ensure program continuation for both the FMNP and NATA. More importantly, we think our collective experience illustrates the value of investing in developing curricula, educational materials, partnerships, and policies that can be readily modified to meet the needs of clientele and that doing so contributes to program resiliency in the face of change.

## CONFLICT OF INTEREST

None declared.

## AUTHOR CONTRIBUTION

**Martin B. Main:** Conceptualization (equal); Methodology (equal); Writing‐original draft (lead); Writing‐review & editing (equal). **Holly K. Ober:** Conceptualization (equal); Methodology (equal); Writing‐original draft (supporting); Writing‐review & editing (equal). **Shelly A. Johnson:** Conceptualization (equal); Methodology (equal); Writing‐original draft (supporting); Writing‐review & editing (equal).

## Supporting information

Appendix S1‐S3

## Data Availability

Program metrics and evaluation data for the Florida Master Naturalist Program (FMNP) and the Natural Areas Training Academy (NATA) used in this paper and a README document are available on DRYAD: https://doi.org/10.5061/dryad.kd51c5b43.
