# Supplementary material for: Resilient structure of nature‐based extension programs facilitates transition to online delivery and maintains participant satisfaction
Source: Ecol Evol. 2020 Oct 27;10(22):12508–14. doi: 10.1002/ece3.6860 (PMC7679548; doi:10.1002/ece3.6860)
Supplement: Supplementary file 1 — Appendix S1‐S3 [file ECE3-10-12508-s001.zip › ece36860-sup-0001-AppendixS1.docx]

**Appendix S1.** Courses offered by the Florida Master Naturalist Program (FMNP) and Natural Areas Training Academy (NATA), course contact hours, course formats, and total course participants during pre-COVID period (2019 and earlier) and during the early COVID period (March 16 - June 30, 2020).

|  |  | **Course hours** | **Pre-COVID Period^a^** | | | | **Early COVID Period** | |
| --- | --- | --- | --- | --- | --- | --- | --- | --- |
| **Course** | **Program** |  | **Course format** | **Participants** | | **Course format** | | **Participants^b^** |
| Coastal Systems | FMNP | 40 | 100% in-person | | 5,857 | Blended (70% online: 30% in-person) | | 20 (100%) |
| Freshwater Systems | FMNP | 40 | 100% in-person | | 4,946 | NA | | 20 (100%) |
| Upland Systems | FMNP | 40 | 100% in-person | | 3,848 | Blended (50% online: 40% in-person) | | 20 (100%) |
| Coastal Shoreline Restoration | FMNP | 24 | 100% in-person | | 388 | Blended (55% online: 45% in-person) | | 39 (95%) |
| Conservation Science | FMNP | 24 | 100% in-person | | 618 | NA | | NA |
| Environmental Interpretation | FMNP | 24 | 100% in-person | | 597 | Blended (85% online: 15% in-person) AND 100% online | | 35 (80%) |
| Habitat Evaluation | FMNP | 24 | 100% in-person | | 514 | Blended (60% online: 40% in-person) | | 14 (70%) |
| Wildlife Monitoring | FMNP | 24 | 100% in-person | | 541 | Blended (65% online: 35% in-person) AND 100% online | | 46 (77%) |
| S-130/190 Basic Wildland Firefighter | NATA | 40 | Blended (20% online: 80% in-person) | | 228 | NA | | NA |
| RX-410 Smoke Management Techniques | NATA | 32 | 100% in-person | | 32 | NA | | NA |
| Collaborating to Manage Ecosystems | NATA | 24 | Blended (33% online: 66% in-person) | | 97 | NA | | NA |
| Conservation Site Assessment & Planning | NATA | 24 | Blended (40% online: 60% in-person) | | 24 | NA | | NA |
| Managing for Diversity | NATA | 24 | Blended (50% online: 50% in-person) | | 61 | NA | | NA |
| Managing Visitors and Volunteers | NATA | 24 | Blended (33% online: 66% in-person) | | 71 | 100% online | | 30 (100%) |
| S-212 Wildland Fire Chainsaws | NATA | 24 | 100% in-person | | 40 | NA | | NA |
| S-215 Fire Ops in the Wildland-Urban Interface | NATA | 16 | 100% in-person | | 105 | NA | | NA |
| S-131 Firefighter Type I | NATA | 12 | 100% in-person | | 109 | 100% online | | 40 (100%) |

^a^Pre-COVID period for FMNP is defined as 2001-2019; NATA is defined as 2015-2019

^b^Participant values represent total FMNP and NATA enrollment for courses initiated during the early-COVID period; values in parentheses (%) represent percent enrollment based on maximum possible enrollment (values may represent more than one course and FMNP course sizes vary)
