# Supplementary material for: Resilient structure of nature‐based extension programs facilitates transition to online delivery and maintains participant satisfaction
Source: Ecol Evol. 2020 Oct 27;10(22):12508–14. doi: 10.1002/ece3.6860 (PMC7679548; doi:10.1002/ece3.6860)
Supplement: Supplementary file 1 — Appendix S1‐S3 [file ECE3-10-12508-s001.zip › ece36860-sup-0003-LegendS2.docx]

Appendix S2. Annual course and enrollment totals for the Florida Master Naturalist Program (FMNP; 2001-2019) and Natural Areas Training Academy (NATA; 2000-2019).
