# Supplementary material for: Resilient structure of nature‐based extension programs facilitates transition to online delivery and maintains participant satisfaction
Source: Ecol Evol. 2020 Oct 27;10(22):12508–14. doi: 10.1002/ece3.6860 (PMC7679548; doi:10.1002/ece3.6860)
Supplement: Supplementary file 1 — Appendix S1‐S3 [file ECE3-10-12508-s001.zip › ece36860-sup-0004-AppendixS3.docx]

**Appendix S3.** Guiding principles followed when developing agendas and managing courses for online courses offered by the Florida Master Naturalist Program (FMNP) and Natural Areas Training Academy (NATA) Extension programs.

**General Guidance throughout course**

- Incorporate opportunities for each participant to synthesize the information learned throughout the course.
- Limit course capacity to a manageable group size that allows for meaningful interaction during live online meetings and ensures instructors can provide individualized feedback on each asynchronous activity or assignment.
- Provide opportunities for course participants to connect with one another during and after the course.

**Synchronous Online Meetings**

- Provide guidance on using video conference technology and dedicate a specific time for participants to “practice” with the online format to ensure they can see, hear, and participate prior to formal group sessions.
- Request that participants log in prior to the official start time for each formal group session to ensure each group session begins on time.
- Include ice breakers and create opportunities for every participant to speak to the entire class to build camaraderie from the onset of the course.
- Explain connections between material covered in the asynchronous activities and the synchronous meetings so it’s clear how the entire experience will advance participant learning.
- Allow time for participants to share the results of their asynchronous activities during online meetings.
- Avoid long lectures during group sessions to prevent participant boredom.
- Incorporate at least one break-out group for every 3 hours of content to stimulate participant engagement.
- Break up longer sessions into bite-sized sections and alternate between passive elements (e.g., lectures) and interactive elements.
- Provide opportunities to allow all participants to learn from discussions had by other groups.
- Incorporate at least one break for every 2 hours of content.
- Incorporate extra time into agendas to avoid exceeding the time by which you have told participants the session will end.

**Asynchronous Activities**

- Self-directed activities should include clear learning objectives and reporting requirements.
- Ensure all participants understand what additional activities are required, and the deadlines.
- Provide clear instructions for participants to complete activities on their own.
- Schedule adequate time for participants to complete the activities prior to the next online meeting.
- If providing pre-recorded presentations, use a variety of presenters to alleviate monotony.
