# Supplementary figures and images for: Resilient structure of nature‐based extension programs facilitates transition to online delivery and maintains participant satisfaction
Source: Ecol Evol. 2020 Oct 27;10(22):12508–14. doi: 10.1002/ece3.6860 (PMC7679548; doi:10.1002/ece3.6860)

### FMNP Course Offerings by Year

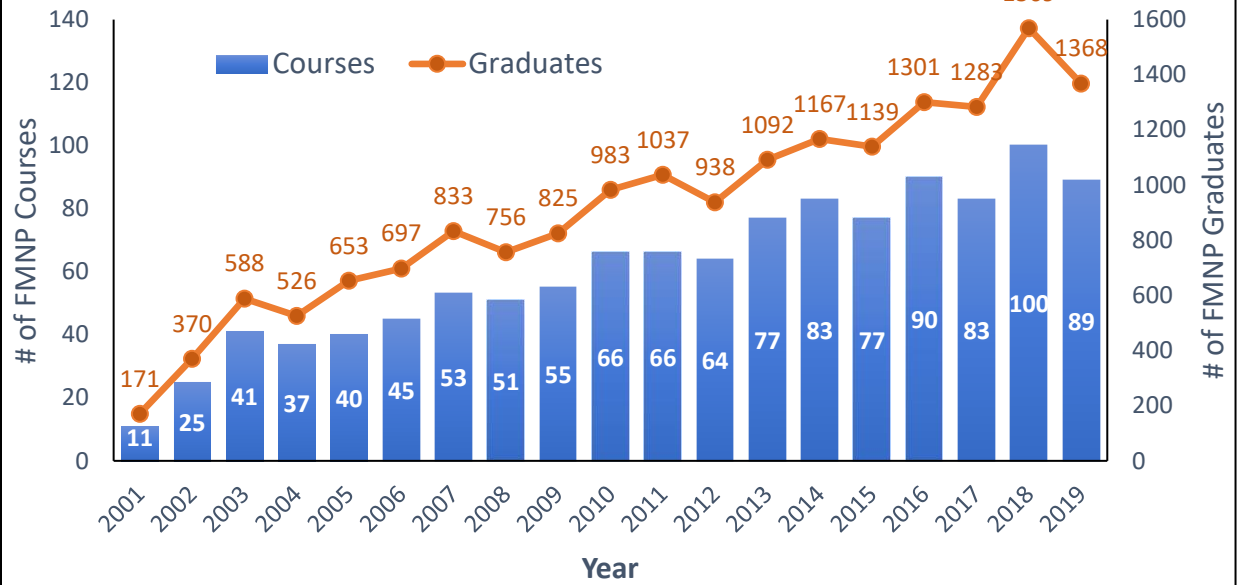

### NATA Course Offerings by Year

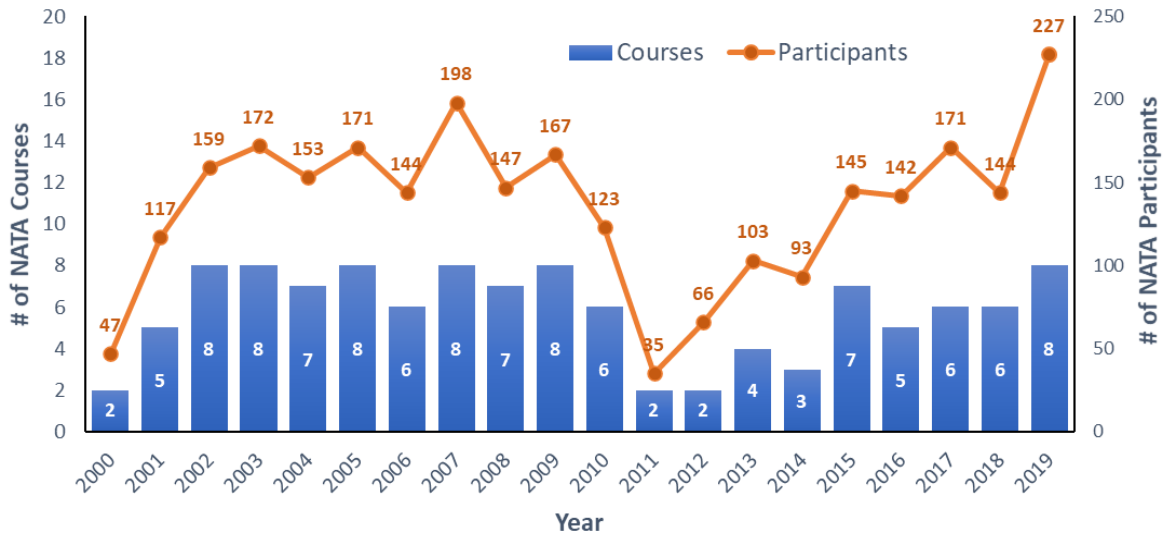

Supplement: Supplementary file 1 — Appendix S1‐S3 [file ECE3-10-12508-s001.zip › ece36860-sup-0002-AppendixS2.pdf]
